# Right Hemi-Diaphragmatic Rupture: An Injury Missed or Masked?

**Published:** 2015-05-01

**Authors:** Manoj Joshi, Anjan Dhua

**Affiliations:** Department of Pediatric Surgery, Pondicherry Institute of Medical Sciences

**Keywords:** Traumatic diaphragmatic rupture, Thoraco-abdominal trauma, Hemothorax

## Abstract

Right sided traumatic diaphragmatic rupture in children is uncommon and may escape early detection. Missed injuries are associated with high mortality and morbidity due to incarceration and strangulation of abdominal viscera. We report a 15-month-old child with blunt trauma chest and abdomen, who presented with bilateral hemothoraces and liver laceration seven days after the incident. Diagnosis of right diaphragmatic rupture was confirmed after another week. The surgical repair of diaphragmatic rupture was undertaken successfully.

## INTRODUCTION

Traumatic diaphragmatic rupture (TDR) is an uncommon and serious injury in children. Right sided TDR is known for being missed in acute settings as solid organ and hollow viscera injury often take precedence in surgical care. [1] However, even in late presentations, diagnosis of right sided TDR is a challenge and diagnostic delay may occur.[2] Since most associated solid organ injuries are managed expectantly, diagnosis of TDR is important for timely surgical intervention. Right sided TDR is also associated with higher mortality and requires more urgent surgical correction. [3] Herein, we describe a child with right sided TDR, who was diagnosed late.

## CASE REPORT

A 15-month-old boy was referred with history of fall from stairs and pressed under the person carrying him. He was initially managed at a local hospital on outdoor basis for difficulty in breathing. After seven days of the accident, he was referred to us because of gradually worsening respiratory distress. On examination, he was alert but irritable, with a heart rate of 120/minute, blood pressure of 90/60 mmHg and respiratory rate of 54/minute with in-drawing of intercostal muscles. His arterial oxygen saturation was 94% at room air. There were no external signs of injury over the chest or abdomen. Air entry was reduced on right side of chest and apex beat was normally placed. Abdominal examination was unremarkable. Chest X-ray was repeated and compared with the one done on the day of accident. Lung fields appeared clear as compared to previous X-ray. Ultrasound abdomen revealed free fluid in peritoneal cavity with laceration of segment VII of the liver. Child was initially managed in intensive care and remained hemodynamically stable, but had tachypnea which was out of proportion to the X-ray findings. His condition worsened further and oxygen requirement to maintain saturation increased.

Contrast enhanced Computerized Tomography (CT) scan thorax and abdomen was done which revealed bilateral massive effusion, more on right side than left. Bilateral chest intubation was done (Fig.1a). The tubes at insertion drained 650 ml and 350 ml of sero-hemorrhagic fluid from right and left hemi-thoraces, respectively and tachypnea gradually normalized.

Left chest tube was removed after three days, but, right tube was continuously draining about 100 ml of serous fluid daily. Repeat chest X-ray showed clear apical lung zones, a well-defined opacity occupying right mid and lower lung zones and ill-defined right dome of diaphragm (Fig.1b). Diaphragmatic injury was suspected and contrast CT scan chest and abdomen was repeated which showed right lobe of liver herniating into thorax through a defect in the dome of right diaphragm along with ipsilateral loculated pneumothorax (Fig.1c,1d).

**Figure:**
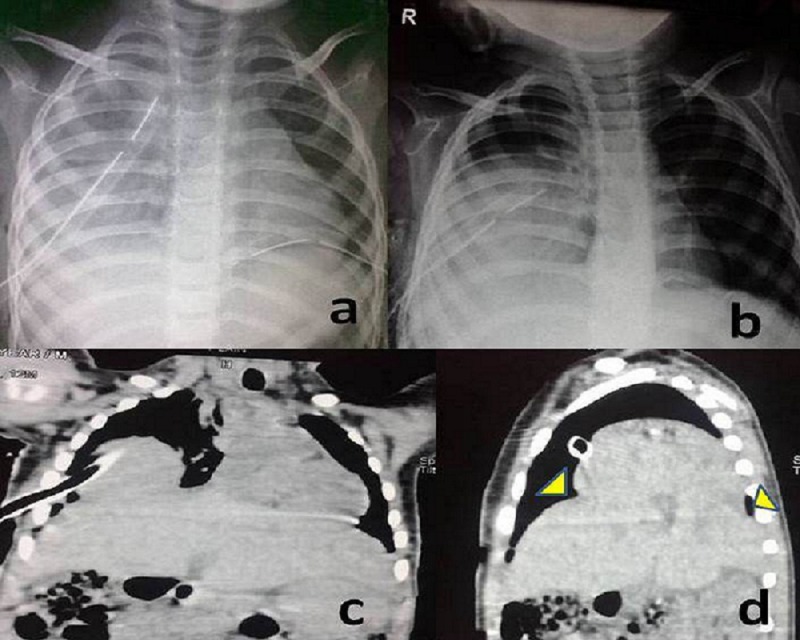
Figure 1: Figure 1: (a) Radiograph - immediately after inserting chest tubes. (b) Persistent radio-opacity in right lower zones after a week of chest tube insertion. (c) Coronal section of CT thorax showing hepatothorax. (d) Sagittal section of CT thorax showing margins of diaphragm around hepatothorax (Arrow heads)

After optimization of his general condition, right posterolateral thoracotomy was done. The intraoperative findings confirmed a large diaphragmatic dome rupture with liver occupying right hemithorax (Fig.2a,2b,2c). The diaphragmatic edges were carefully mobilized off the lacerated liver surface and repaired by non-absorbable suture. Subsequently patient had an uneventful recovery. After 10 months of follow-up, the patient is asymptomatic.

**Figure:**
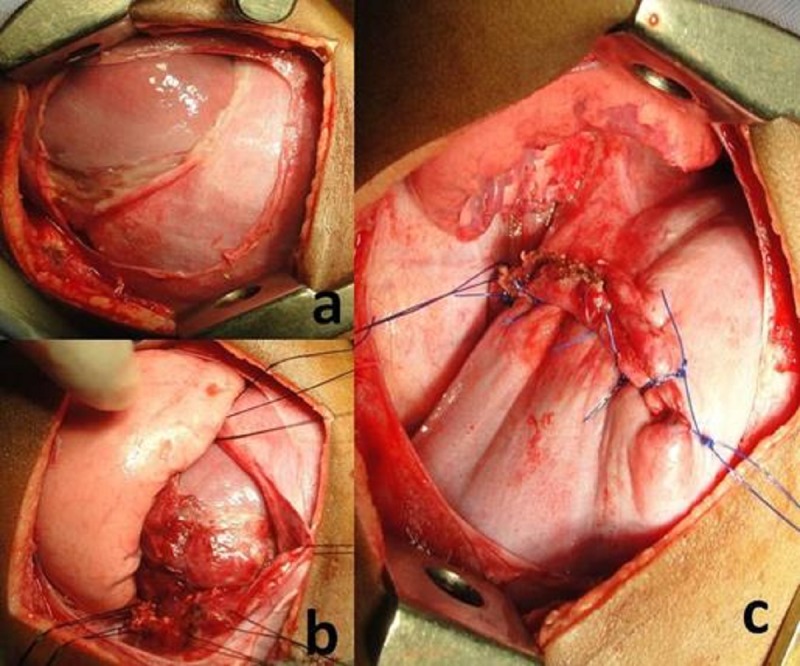
Figure 2: (a) Intra-operative image - liver across the rent into thoracic cavity. (b) The lacerated liver surface with the edges of the rent defined with stay sutures. (c) Rent repaired primarily.

## DISCUSSION

In acute phase, right sided TDR in children often remains unrecognized in 10-50% of cases.[4] The initial radiograph and CT scan may not be helpful. The injury is usually missed in approximately 50% of cases.[2,5,6] Massive effusion may conceal the underlying TDR by non-visualization of diaphragmatic rim on X-ray. Even in late presentations, unless there’s visceral herniation, an injury may easily be missed even on CT scan because of the thin diaphragmatic rim. Same happened in our case and a diagnostic delay occurred. A situation wherein the TDR is masked by positive pressure ventilation and manifested clinically when disconnected from the ventilator, has also been described in the literature.[7] Rarely, there could be delayed rupture of devitalized tissue also.[8]

In index case the diaphragmatic injury was not reported initially on X ray possibly because liver was positioned below the diaphragm. After the chest tube drainage, the resultant negative intra-thoracic pressure gradient would have made hepatothorax and so on repeat CT scan it was picked up.

A partial resolution of the right sided hemothorax was seen in the initial chest X-ray in our case. This could possibly be explained by fluid gradually percolating into the peritoneal cavity across the diaphragmatic tear. This sign must be viewed with caution. Another important feature in our case was persistent serous drainage in the right chest tube which prompted us for a repeat CT scan.

Adhesions may be formed between margins of ruptured diaphragm and herniated liver in delayed presentations. Repair of large right sided defect with liver tear is technically difficult by laparotomy which needs mobilization of friable lacerated liver tissue to repair the defect. An open thoracotomy, therefore, is a convenient approach for repair if there is no associated strangulation or perforation injury of bowel.[8] Thoracoscopic repair has been described if there is no associated abdominal organ injury.[9]

Based upon our experience it is suggested that right sided diaphragmatic rupture must be considered in a child with thoraco-abdominal injury. Patient may present late and CT scan may miss the diagnosis initially if there is no hepatothorax. High index of suspicion is thus needed specially in children with blunt thoraco-abdominal trauma with pleural effusion and persistent serous effluent in the chest tube. Serial radiographs and repeat CT scan after intercostal drainage, helps in early diagnosis of this serious injury.

## Footnotes

**Source of Support:** Nil

**Conflict of Interest:** None declared

